# Discrepancy of echocardiography and computed tomography in initial assessment and 2-year follow-up for monitoring Marfan syndrome and related disorders

**DOI:** 10.1038/s41598-022-19662-y

**Published:** 2022-09-12

**Authors:** Nick Lasse Beetz, Tobias Daniel Trippel, Karla Philipp, Christoph Maier, Thula Walter-Rittel, Seyd Shnayien, Petra Gehle

**Affiliations:** 1 Department of Radiology, Charité – Universitätsmedizin Berlin, Corporate Member of Freie Universität Berlin and Humboldt-Universität Zu Berlin, Augustenburger Platz 1, 13353 Berlin, Germany; 2 Department of Internal Medicine – Cardiology, Charité – Universitätsmedizin Berlin, Corporate Member of Freie Universität Berlin and Humboldt-Universität zu Berlin, Berlin, Germany; 3 DZHK (German Center for Cardiovascular Research), Partner Site Berlin, Berlin, Germany; 4 BIH Biomedical Innovation Academy, Berlin Institute of Health at Charité – Universitätsmedizin Berlin, Berlin, Germany

**Keywords:** Cardiology, Medical research

## Abstract

Patients with Marfan syndrome and related disorders are at risk for aortic dissection and aortic rupture and therefore require appropriate monitoring. Computed tomography (CT) and transthoracic echocardiography (TTE) are routinely used for initial diagnosis and follow-up. The purpose of this study is to compare whole-heart CT and TTE aortic measurement for initial work-up, 2-year follow-up, and detection of progressive aortic enlargement. This retrospective study included 95 patients diagnosed with Marfan syndrome or a related disorder. All patients underwent initial work-up including aortic diameter measurement using both electrocardiography-triggered whole-heart CT and TTE. Forty-two of these patients did not undergo aortic repair after initial work-up and were monitored by follow-up imaging within 2 years. Differences between the two methods for measuring aortic diameters were compared using Bland–Altman plots. The acceptable clinical limit of agreement (acLOA) for initial work-up, follow-up, and progression within 2 years was predefined as <  ± 2 mm. Bland–Altman analysis revealed a small bias of 0.2 mm with wide limits of agreement (LOA) from + 6.3 to − 5.9 mm for the aortic sinus and a relevant bias of − 1.6 mm with wide LOA from + 5.6 to − 8.9 mm for the ascending aorta. Follow-up imaging yielded a small bias of 0.5 mm with a wide LOA from + 6.7 to − 5.8 mm for the aortic sinus and a relevant bias of 1.1 mm with wide LOA from + 8.1 to − 10.2 mm for the ascending aorta. Progressive aortic enlargement at follow-up was detected in 57% of patients using CT and 40% of patients using TTE. Measurement differences outside the acLOA were most frequently observed for the ascending aorta. Whole-heart CT and TTE measurements show good correlation, but the frequency of measurement differences outside the acLOA is high. TTE systematically overestimates aortic diameters. Therefore, whole-heart CT may be preferred for aortic monitoring of patients with Marfan syndrome and related disorders. TTE remains an indispensable imaging tool that provides additional information not available with CT.

## Introduction

Marfan syndrome is the most common genetic disorder affecting the aorta with an estimated incidence of 2–3 per 10,000 individuals. Less common inherited connective tissue disorders of the aorta include Loeys-Dietz syndrome and the vascular type of Ehlers-Danlos syndrome (EDS)^1^. Mutations underlying classic Marfan syndrome involve the *FBN1* gene, whereas mutations in *TGFBR1* and *2, SMAD2* and *3,* and *TGFB2* and *3* can be detected in Loeys-Dietz syndrome types 1 to 6, and a *Col3A1* gene mutation has been described for the vascular type of EDS^2,3^. Patients with untreated genetic aortic disorders have a dramatically lower life expectancy as they are at risk for aortic dissection, aortic rupture, and heart failure^4–6^. Therefore, aortic monitoring is of great importance to reduce morbidity and mortality in these patients.

International guidelines recommend imaging of the aorta for initial aortic work-up and follow-up of patients with proven or suspected genetic aortic disease^7,8^. Correct measurement of aortic diameters is essential for risk stratification and preoperative evaluation before surgical repair. The 2017 ESC guideline states that surgery is indicated in all patients with Marfan syndrome and a maximal aortic diameter ≥ 50 mm, and that in patients with additional risk factors and in patients with a *TGFBR1* or *TGFBR2* mutations surgery should be considered at a maximal aortic diameter ≥ 45 mm^9^. One of these risk factors is as an increase in aortic diameter on repeated measurements using the same imaging technique. Therefore, correct measurement is also important during follow-up imaging to detect patients with progressive aortic enlargement. The availability of cardiovascular imaging techniques has greatly improved long-term survival especially in Marfan syndrome^10^.

The most commonly used imaging modalities for aortic monitoring are computed tomography (CT) and transthoracic echocardiography (TTE). Alternatively, magnetic resonance imaging (MRI) can be performed, though its use is often reserved for younger patients due to higher cost, longer examination time, and limited availability^11^. As measurement conventions of aortic diameters differ between institutions and imaging modalities, patients with genetic aortic disease should undergo follow-up imaging preferably at the same institution using the same imaging modality, especially if previous cross-sectional imaging did not correlate sufficiently^12^. The guideline on aortic disease endorsed by the American College of Cardiology (ACC) and the American Heart Association (AHA) recommends measurement of the external aortic diameter perpendicular to the axis of blood flow when CT or MRI is used and measurement of the internal aortic diameter perpendicular to blood flow when TTE is used^8,13^. There are three typical CT measurements of the aortic root: aortic annulus, aortic sinus and sinotubular junction. The ACC/AHA guideline states that the widest diameter, typically at the mid-sinus level, should be used. This CT measurement of the aortic root has shown to have the best reproducibility^14^.

State-of-the-art CT scanners allow ECG-triggered aortic imaging with high diagnostic accuracy. Hence, CT is the modality of choice for preoperative imaging in patients scheduled for transcatheter structural heart interventions and has been used as the “gold standard” in earlier comparative studies^15,16^. Exact measurement of aortic diameters is crucial for both initial work-up and follow-up monitoring, as patients with aortic aneurysm (defined by these measurements) and progressive aortic enlargement are at risk for life-threatening aortic rupture and dissection^17^.

In patients with genetic aortic disease, detecting progressive enlargement of the aorta is extremely important as they may require surgical aortic repair^18^. Even if follow-up imaging is performed at a different institution with different measurement conventions, progressive aortic enlargement can be detected more objectively if prior CT scans are provided to the radiologist rather than TTE, which has larger inter- and intraobserver variability^19^.

This study aims at comparing state-of-the-art whole-heart CT and TTE aortic measurements for initial work-up, follow-up, and detection of progressive aortic enlargement in patients with Marfan syndrome or a related disorder.

## Materials and methods

### Study design

The study was approved by the institutional review board (Ethikkommission der Charité – Universitätsmedizin Berlin), which waived the need for patient consent. In this single-center comparative cohort study, we compared measurements of aortic diameters in a retrospective dataset of patients who underwent CT and TTE of the aorta. In a subgroup of patients who did not undergo surgical repair and were monitored by follow-up aortic imaging within 2 years, we analyzed measurement accuracy by comparing measurements taken at initial work-up and follow-up and evaluated increases in aortic diameters between the two time points as a measure of disease progression. The study was in compliance with the Declaration of Helsinki.

### Patient population and characteristics

We enrolled patients diagnosed with Marfan syndrome or a related disorder including Loeys-Dietz syndrome, Ehlers-Danlos syndrome, and familial aortic dissection at the outpatient Marfan Center of Charité University Hospital in Berlin. Marfan syndrome was diagnosed using the 2010 revised Ghent Criteria, which are based on physical examination, aortic imaging, family history, and genetic testing in some cases^4^. The diagnosis of Marfan syndrome requires presence of clinical criteria including aortic root dilatation/dissection and ectopia lentis. If genetic testing was performed, the presence of *FBN1* mutation was confirmed in all patients diagnosed with Marfan syndrome. Patients diagnosed with Loeys-Dietz syndrome or Ehlers-Danlos syndrome had undergone genetic testing, which confirmed typical mutations.

All patients were referred to the Department of Radiology between 2015 and 2020. All patients underwent ECG-triggered CT angiography of the aorta and TTE of the proximal thoracic aorta for initial assessment. Our additional follow-up analysis was performed in a subgroup of patients who did not undergo surgical treatment within 2 years after initial work-up. Instead, the subgroup was monitored and underwent follow-up imaging with both CT and TTE due to suspected progression of aortic enlargement, suspected aortic dissection, or preoperative evaluation for surgical repair or replacement. Exclusion criteria were prior interventional or surgical aortic repair. As aortic enlargement may be due to causes other than Marfan syndrome or a related disorder, patients aged above 55 years were excluded from this study.

### Image acquisition and aortic diameter measurement

**Figure 1:**
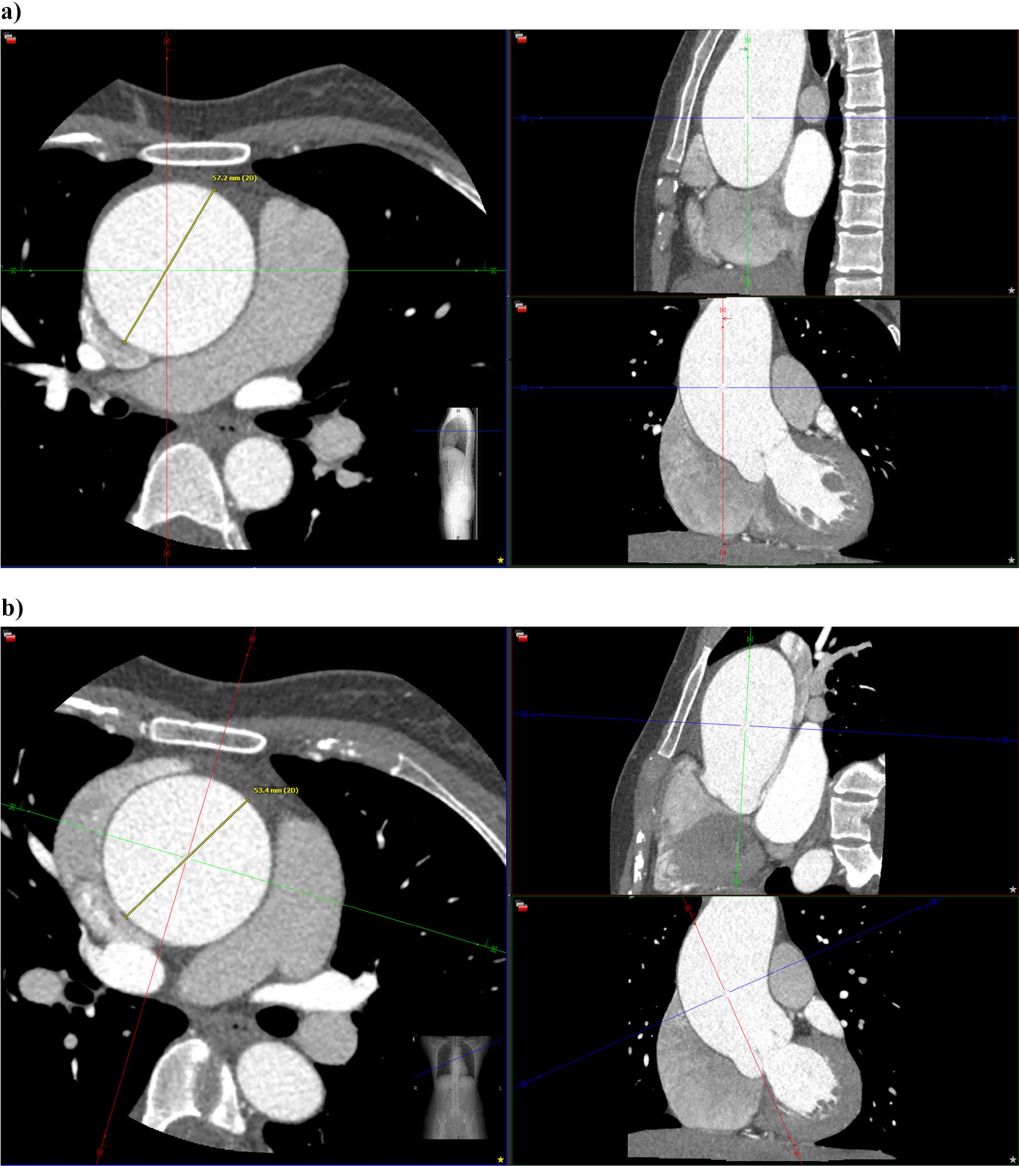
Example illustrating the importance of double oblique multiplanar reconstruction perpendicular to the course of the vessel for correct aortic diameter measurement: (**a**) from unreconstructed CT, a wrong ascending aortic diameter of 57.2 mm is measured; (**b**) measurement from reconstructed CT yields the correct aortic diameter of 53.4 mm. Like TTE, unreconstructed CT may preclude correct diameter measurement perpendicular to the course of the vessel in patients with an elongated aorta and/or thoracic deformities. CT = computed tomography; TTE = transthoracic echocardiography.

All patients referred to the Department of Radiology were examined in the same single-source 256-row CT scanner (Revolution CT, General Electric, Milwaukee, USA). Intravenous bolus injection of iodinated contrast medium was administered. In diastole an axial ECG-triggered whole-heart scan including the aortic root was obtained, directly followed by a helical scan of the entire aorta. Bolus tracking with SmartPrep (General Electric, Milwaukee, USA) was used to ensure adequate opacification of the aorta. Aortic diameters were determined from double oblique multiplanar reconstructions perpendicular to the course of the vessel^20^. All measurements were taken at the levels of the aortic sinus and the largest diameter of the ascending aorta (between aortic arch and sinotubular junction) using Visage (Visage Imaging, Berlin, Germany). At the level of aortic sinus, the maximal (non-anatomically landmarked) diameter at the mid-sinus level was used as recommended by the ACC/AHA guideline^8^. During serial follow-up imaging, aortic diameter was consistently measured from outer edge to outer edge. All measurements were performed by the same radiologist with more than 7 years of experience in cardiovascular imaging. An example illustrating aortic diameter measurement from double oblique multiplanar CT reconstructions perpendicular to the course of the aorta is shown in Fig. 1.

All patients also underwent two-dimensional TTE with the Epic 700 cardiac ultrasound machine (Philips, Amsterdam, the Netherlands) at initial work-up and at follow-up. Aortic diameters at the aortic sinus and the ascending aorta were measured in diastole from inner edge to inner edge. All examinations and measurements were performed by an attending-level cardiologist and an attending-level cardiac surgeon, both board-certified, with over 20 years of experience in echocardiography, using an established and standardized protocol based on recent echocardiography guidelines^21^.

### Comparison of measurement methods

Pearson’s correlation coefficient was used to measure correlation between CT and TTE. For further analysis, Bland–Altman plots were used to compare the two measurement methods regarding initial work-up, follow-up, and change in diameter over time. The acceptable clinical limit of agreement for the difference between the two methods for initial work-up, follow-up, and progression within 2 years was predefined as <  ± 2 mm. Diameters of the aortic sinus and ascending aorta obtained by CT- and TTE-based measurement were compared for initial work-up and follow-up. Measurement differences showing an increase in aortic diameters between initial and follow-up imaging were compared between CT and TTE using Bland–Altman plots.

### Statistical analysis

All data analyses were performed using IBM SPSS Statistics version 27 (IBM, Armonk, New York, USA).

## Results

### Baseline data

A total of 95 patients with a mean age of 35 ± 10 years were included in this study, among them 60 men (63%) and 35 women (37%). A majority of 77 patients (81%) were diagnosed with Marfan syndrome, 10 patients (10%) with Loeys-Dietz syndrome, 6 patients (6%) with Ehlers-Danlos syndrome, and 2 patients (2%) with familial aortic dissection at the outpatient Marfan Center of Charité University Hospital in Berlin. For initial work-up, all 95 patients underwent aortic imaging with CT and TTE. In a subgroup of 42 patients (44%) who did not undergo aortic repair within 2 years and were instead monitored by follow-up imaging, aortic diameters were measured again with both CT and TTE. The mean interval between initial and follow-up imaging was 581 ± 170 days. Seventy-nine patients (83%) were on medication including a renin-angiotensin system antagonist or a beta-blocker, whereas 16 patients (17%) did not receive any medication. The mean dose-length product (DLP) of the aortic CT scan was 502 ± 193 mGy * cm. Baseline data are summarized in Table 1.

**Table 1:** Baseline data of patients included in this study.

	Total (n = 95)
Age, years*	35 ± 10
**Genetic aortic disease, n (%)**	
Marfan syndrome	77 (81%)
Loeys-Dietz syndrome	10 (10%)
Ehlers-Danlos syndrome	6 (6%)
Familial aortic dissection	2 (2%)
Female sex, n (%)	35 (37%)
**Medication, n (%)**	
RAAS or beta-blockers	78 (83%)
None	16 (17%)
DLP, mGy * cm	502 ± 193

RAAS, renin–angiotensin–aldosterone system; DLP, dose-length product.

### Diameters of aortic sinus and ascending aorta

At initial work-up, the mean diameter of the aortic sinus was 41.1 ± 5.9 mm using CT and 41.0 ± 5.3 mm using TTE. The mean diameter of the ascending aorta was 33.9 ± 7.3 mm using CT and 35.6 ± 7.3 mm using TTE.

At follow-up, the mean diameter of the aortic sinus was 43.9 ± 5.0 mm using CT and 43.4 ± 5.6 mm using TTE. The mean diameter of the ascending aorta was 37.3 ± 8.0 mm using CT and 38.4 ± 9.4 mm using TTE.

### Progressive aortic enlargement

In the subgroup of 42 patients who underwent follow-up imaging, progressive aortic enlargement was defined as an increase in the diameter of the aortic sinus or the ascending aorta of at least 2 mm within 2 years. Follow-up aortic imaging for monitoring patients with Marfan syndrome or a related disorder detected progressive aortic enlargement in 24 patients (57%) using CT versus 17 patients (40%) using TTE. In these patients, we found a mean increase in aortic diameter of 2.6 ± 1.1 mm using CT and of 3.8 ± 2.8 mm using TTE.

### Pearson’s correlation between CT and TTE measurement

The two measurement methods, CT and TTE, showed statistically significant correlation. For initial measurement, Pearson’s correlation coefficients were 0.85 for the aortic sinus and 0.87 for the ascending aorta. For follow-up measurement, Pearson’s correlation coefficients were similar with 0.83 for the aortic sinus and 0.87 for the ascending aorta. All measurement correlations were statistically significant at the *p* < 0.01 level.

### Diameters of the aortic sinus: comparison of measurement methods

**Figure 2:**
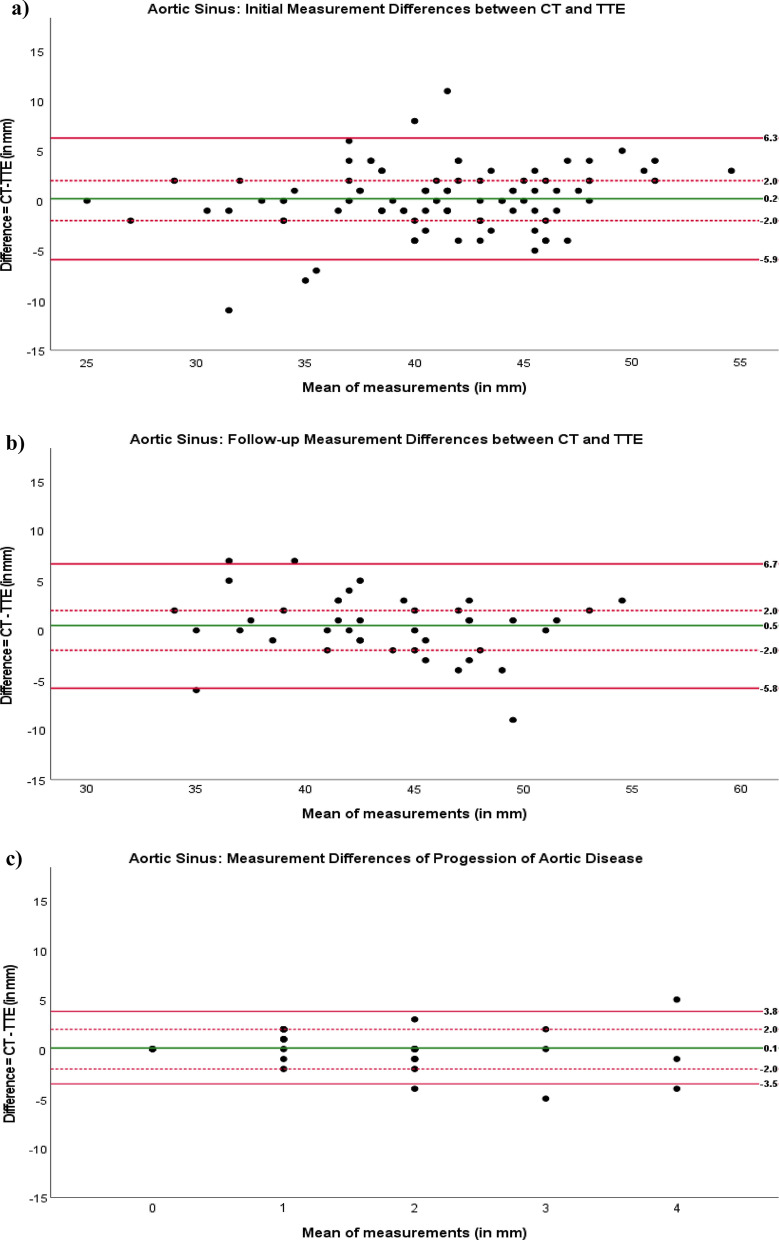
Bland–Altman plots for analysis of measurement differences in aortic sinus diameter in patients with Marfan syndrome or a related disorder for (**a**) initial work-up, (**b**) follow-up, and (**c**) progression of aortic disease. Red lines: upper and lower limits of agreement. Dotted red line: clinically acceptable limit of agreement for difference. Green line: agreement bias.

For initial measurement of the aortic sinus, Bland–Altman analysis showed a small discrepancy of the two measurement methods with a bias of 0.2 mm. There were wide limits of agreement ranging from + 6.3 to − 5.9 mm. Thirty-two patients (34%) were outside the clinically acceptable limits of agreement regarding the difference between the two measurement methods (> 2 mm), whereas 63 patients (66%) were within the limits of agreement (Fig. 2a).

For the subgroup of patients who underwent follow-up aortic imaging, Bland–Altman analysis showed a small discrepancy of the two measurement methods with a bias of 0.5 mm. There were wide limits of agreement ranging from + 6.7 to − 5.8 mm. Sixteen patients (38%) were outside the clinically acceptable limits of agreement regarding the difference between the two measurement methods (> 2 mm), whereas 26 patients (62%) were within the limits of agreement (Fig. 2b).

Bland–Altman plots analyzing differences in progression of aortic enlargement from initial work-up and follow-up showed a bias of 0.1 mm with wide limits of agreement ranging from + 3.8 to -3.5 mm. Five patients (12%) were outside the clinically acceptable limits of agreement regarding this parameter (Fig. 2c).

### Diameters of the ascending aorta: comparison of measurement methods

**Figure 3:**
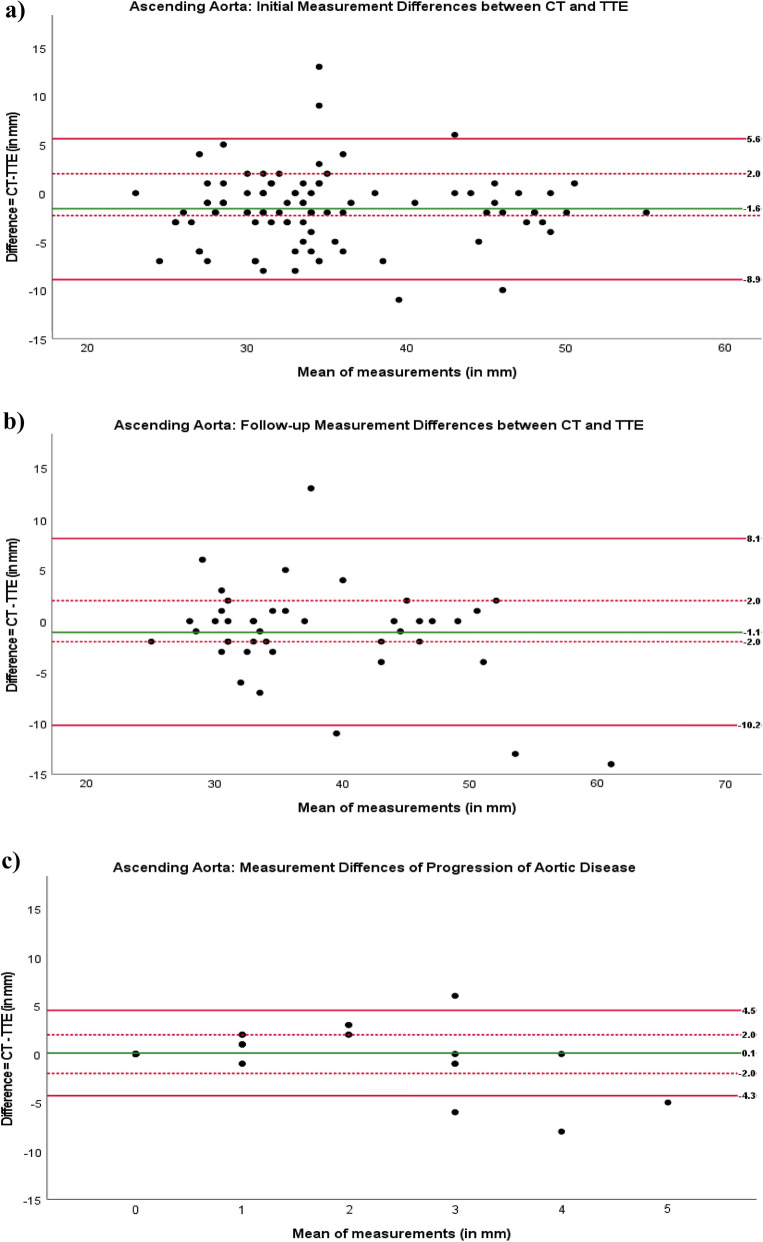
Bland–Altman plots for analysis of measurement differences in ascending aorta diameter in patients with Marfan syndrome or a related disorder for (**a**) initial work-up, (**b**) follow-up, and (**c**) progression of aortic disease. Red lines: upper and lower limits of agreement. Dotted red line: clinically acceptable limit of agreement for difference. Green line: agreement bias.

For initial measurement of the ascending aorta, Bland–Altman analysis showed a relevant discrepancy of the two measurement methods with a bias of − 1.6 mm. There were wide limits of agreement ranging from + 5.6 to − 8.9 mm. Thirty-eight patients (40%) were outside the clinically acceptable limits of agreement for the difference (> 2 mm) between the two measurement methods, whereas 58 patients (60%) were within the limits (Fig. 3a).

For the subgroup of patients who underwent follow-up aortic imaging, Bland–Altman analysis showed a relevant discrepancy of the two measurement methods with a bias of 1.1 mm. There were wide limits of agreement ranging from + 8.1 to − 10.2 mm. Fifteen patients (36%) were outside the clinically acceptable limits of agreement for the difference (> 2 mm) between the two measurement methods, whereas 27 patients (64%) were within the limits (Fig. 3b).

Bland–Altman plots analyzing measurement differences regarding progressive aortic disease showed a bias of 0.1 mm with wide limits of agreement ranging from + 4.5 to − 4.3 mm. Thirteen patients (31%) were outside the clinically acceptable limits of agreement for the difference (Fig. 3c).

## Discussion

In this study we analyzed differences in aortic diameter measurement using ECG-triggered whole-heart CT and TTE in patients with Marfan syndrome or a related disorder for initial work-up, follow-up, and detection of increases in aortic diameter within 2 years. Even though the two measurement methods showed good correlation and small overall differences of mean, Bland–Altman plots revealed that the individual differences in aortic diameters measured using CT and TTE show wide limits of agreement in both initial work-up and follow-up, especially for measurement of ascending aortic diameter. For both initial and follow-up aortic imaging, Bland–Altman analysis exposed a relevant measurement bias regarding the ascending aorta. TTE detected fewer cases of progressive aortic enlargement within 2 years of initial measurement compared with CT. For both the ascending aorta and the aortic sinus, we found high rates of measurement differences outside the acceptable clinical limits of agreement for differences in initial work-up, follow-up, and identification of increases in aortic diameter.

Imaging of the aorta using TTE is widely available at relatively low cost and offers the additional benefit of allowing assessment of cardiac function. For example, echocardiography provides information on the severity of mitral valve prolapse and aortic regurgitation, which is common in Marfan syndrome ^22^. However, accurate aortic diameter measurement might be hampered by thoracic deformity and an elongated ascending aorta^23^. Another disadvantage of TTE compared to CT is that only the aortic root and ascending aorta are visualized, meaning no information on the descending aorta can be obtained.

A disadvantage of using CT for aortic imaging is radiation exposure. Patients with Marfan syndrome or a related disorder require aortic imaging for initial work-up and serial follow-up monitoring, ultimately leading to a cumulative dose of potentially harmful ionizing radiation in often young patients. However, average radiation dose for aortic imaging has substantially decreased with state-of-the-art CT scanners and adjusted CT protocols^24,25^. Additionally, adverse effects related to intravenous contrast medium administration can occur, even though severe reactions have been reported in less than 0.01% of cases^26^.

Enlargement of the aortic root is a major criterion for diagnosing genetic aortic disease and is associated with a high risk of aortic dissection and hence cardiovascular mortality^2,4,27^. Life expectancy of patients with Marfan syndrome or a related disorder has improved to near-normal due to better monitoring including aortic imaging and timely surgery^28,29^. Given the increased cardiovascular mortality and morbidity, aortic repair is usually recommended in patients with progression of aortic enlargement^30,31^.

Accurate measurement of aortic diameters is of utmost importance for patients diagnosed with Marfan syndrome or a related disorder because aortic size and progressive aortic enlargement are crucial parameters for predicting fatal rupture of thoracic aortic aneurysm or dissection^32,33^. CT is the modality of choice for measuring aortic diameters for preoperative or preinterventional planning as it is widely available and has proven to be highly accurate^34,35^. Several studies have shown that, while adequate monitoring of the aortic root is also possible with TTE or MRI, CT is superior in detecting aortic dissection^36^. Another advantage of ECG-triggered CT is the option to rule out significant coronary artery disease, which is relevant for preoperative planning^37^. Moreover, additional information including CT body composition parameters relevant for prediction of progressive aortic enlargement and further manifestations of Marfan syndrome or related disorders can be derived^20^.

All aortic measurements during both initial work-up and follow-up were taken by the same observer, as intraobserver variability of aortic measurement is low while interobserver variability may be relevant^38^. Our results show relevant measurement differences between CT and TTE with a relevant bias regarding the ascending aorta. We think this finding is most likely attributable to systematic overestimation of aortic diameters when TTE is used. In most cases in which TTE shows measurement differences outside the acceptable clinical limit of agreement, CT reveals typical thoracic deformity and/or an elongated ascending aorta, which may preclude adequate aortic diameter measurement perpendicular to the axis of blood flow using TTE. In CT, aortic diameters are determined from double oblique multiplanar reconstructions perpendicular to the course of the aorta. Moreover, TTE-based measurement of the aortic sinus may falsely include an ectatic proximal coronary artery—a common phenomenon in patients with Marfan syndrome or a related disorder^39^.

Previous studies have compared CT and TTE measurements of aortic diameter. Bons et al. conclude that there may be large measurement differences in the ascending aorta and therefore also opt for CT or MR angiography^40^. In contrast, a retrospective comparison performed by Ocak et al. suggests that TTE yields substantially lower or even normal diameter measurements when CT demonstrates a dilated aortic root^41^, while Frazo et al. report that TTE underestimates aortic root diameters compared with CT^42^. Unlike our study, the investigators just quoted did not measure aortic diameters in patients with Marfan syndrome and related disorders. Ascending aortic aneurysms in patients with Marfan syndrome have been studied by Amsallem et al. who showed that CT measurements should be obtained on strict transverse plane instead of on three-cavity view or left ventricle-aorta view^43^. Even though we agree that the transverse plane should be used, their conclusion was based on overall differences of the mean which is an important limiting factor for comparing two measurement methods. As a good example in our study the mean diameter of the aortic sinus was 41.1 mm using CT and 41.0 mm using TTE at initial work-up. However, Bland–Altman plots revealed important individual measurement differences which are very relevant in a potentially life-threatening disease. Similarly, on the basis of overall differences of the mean Rodríguez-Palomares et al. concluded that the leading edge to leading edge convention should be used if TTE was compared to CT or MRI^44^. Both studies have used internal diameters for CT measurements, whereas we have used external diameters as recommended by the AHA/ACC guideline^8,13^.

Despite its limited availability, MRI has proven to be an important alternative in several studies showing good correlation with TTE and CT measurements^45^. Especially in young patients with suspected genetic aortic disease, MRI is a good modality for aortic imaging^46^. However, MRI is inferior to CT in terms of accurate multiplanar reconstruction and is less well established in the preoperative or preinterventional assessment of the ascending aorta^47^. Especially in patients with Marfan syndrome or a related disorder, even small differences in aortic diameters may have significant implications for clinical management^48^. Unlike the above-quoted earlier studies, we did not only perform correlation analyses because a high correlation coefficient alone does not automatically mean that there is good agreement between two methods^49^. Instead, by additionally using Bland–Altman plots, we here for the first time show true measurement differences between CT and TTE in patients with Marfan syndrome or a related disorder, who require particularly accurate monitoring.

As the ESC guideline for the management of valvular heart disease states that aortic repair should be considered in patients with Marfan syndrome and risk factors (family history of dissection, size increase of > 3 mm/year in serial examinations), we think that, to ensure robust aortic monitoring, measurement differences at the aortic root should be below 3 mm^9^. However, reliable measurement of aortic diameter using CT comes with a price: radiation in a typically young patient population. State-of-the-art CT scanners have helped to reduce the effective dose of CT angiographies considerably^50^. Considering the risk of rupture and dissection in case of undetected aortic enlargement, the benefits of CT outweigh the risks of radiation exposure even in younger patients. In our study, mean DLP was comparatively low for a potentially life-threatening disease^51^. The high rate of measurement differences between CT and TTE outside the clinically acceptable limits of agreement for initial work-up, follow-up, and detection of progressive disease in serial imaging suggests that the benefit of obtaining more accurate measurements with ECG-triggered CT might justify radiation exposure.

## Conclusion

Highly accurate measurement of aortic diameters in patients at risk for fatal aortic rupture and dissection is warranted. CT and TTE measurements show good correlation, but the frequency of measurement differences outside the acceptable clinical limit of agreement is high, and TTE systematically overestimates aortic diameters. Therefore, monitoring of patients with Marfan syndrome and related disorders may preferably be done using ECG-triggered CT of the aorta for initial work-up, follow-up, and detection of aortic diameter changes over time to ensure reliable measurement for detection of aortic enlargement and identification of progressive disease. Nevertheless, TTE remains an indispensable imaging tool that provides additional information not available with CT.

## Limitations

Our study is limited by the use of a retrospective dataset. Analysis of follow-up aortic measurement was performed in a relatively small subgroup because only a few patients undergo repeated CT imaging before surgery. As this study only included patients who did not yet have aortic repair, there might have been a selection bias toward patients with less severe disease. Finally, even though CT is widely considered the “gold standard” in clinical routine, even CT-based measurements are only approximations of true aortic diameters.

## Data Availability

The datasets generated and/or analyzed during the current study are not publicly available because they include confidential data pertaining to human subjects but are available from the corresponding author on reasonable request.

## Abbreviations

ACC: American College of Cardiology
acLOA: Acceptable clinical limit of agreement
AHA: American Heart Association
CT: Computed tomography
*Col3A1*: Gene encoding for type III collagen
ECG: Electrocardiography
EDS: Ehlers-Danlos syndrome
*FBN1*: Gene encoding for fibrillin-1
LOA: Limits of agreement
MRI: Magnetic resonance imaging
*SMAD2/3*: Gene encoding for mothers against decapentaplegic homolog 2/3
TTE: Transthoracic echocardiography
*TGFB2/3*: Gene encoding for transforming growth factor beta 2/3
*TGFBR1/2*: Gene encoding for transforming growth factor beta receptor I/II
